# Prospectus

**Published:** 1839-06

**Authors:** Eleazar Parmly, Elisha Baker, Solyman Brown

**Affiliations:** Publishing Committee. *New-York*; Publishing Committee. *New-York*; Publishing Committee. *New-York*


### PROSPECTUS.

The plan of "The American Journal of Dental Science," may be expressed in general terms, by saying,—That it is intended as a vehicle of useful information to the numerous Operative Dentists in the United States. That medical gentlemen will be its patrons to some extent, there is little doubt; and that some general readers will become subscribers to the work, and peruse it with interest and profit, we think is equally certain.

The detail of our plan may be seen in the following particulars:

1. The Journal will consist of forty-eight octavo pages per number; twenty-four of which will be employed in re-publishing Standard Works on Dental Theory and Practice; as in the present number.
2. This Specimen Number is a sample of the entire Work as regards the size and quality of the paper; the character of the typography, and the general appearance of the Journal.
3. The re-published Works will be so printed that the numbers may be separated, and the two halves distinctly bound; making two entire works of which the re-publication will amount to at least a volume in each year, worth of itself the whole subscription price of the magazine.
4. Each number will contain the Biography of some Dental Writer or Practitioner, with Portraits in all cases in which they can be obtained.
5. Each number will contain a Review of some Dental Work, or translation from foreign languages.
6. Each number will contain Communications from contributors, on the various topics of useful discussion, relating to the physiology, pathology, preservation, restoration, and general management of the teeth, and adjacent parts.
7. Curious Facts, and Dental Anecdotes, will give interest to the pages of the Journal.
8. The Arts of Quackery will be boldly exposed; and the public will be instructed how to avoid the impositions of ignorant pretenders.
9. Numerous wood cuts, and other prints, will embellish and illustrate the work, as the state of the subscription will justify.
10. The Journal will be published simultaneously in Philadelphia, Baltimore, New-York and Boston, on the first day of every month, to commence as soon as the number of subscribers will permit.
11. Each and every Article admitted to the pages of this Journal, will be accompanied by the name of its author.
12. All Letters and Packages must be post-paid.

13. Any individual who will remit to the Editors, Publishing Committee, or Treasurer, the sum of Five Dollars, shall receive two copies of the Journal through twelve numbers.

14. The work will be Edited by Chapin A. Harris of Baltimore, and Eleazar Parmly of New-York.

15. The Publishing Committee are E. Parmly, E. Baker, and S. Brown, of New-York.

16. Mr. Jahial Parmly of New-York, is the Treasurer of the Association, and will receive subscriptions by mail or otherwise.

17. Subscribers who desire that their numbers should be forwarded otherwise than by mail, will please to give written instructions on the subject.

18. The Journal will be mailed in the four cities of Philadelphia, Baltimore, New-York, and Boston, for subscribers living near those cities respectively.

19. A list of subscribers will be published in at least two numbers of the Journal in each year, and to this end subscribers are requested to give their names in full, with places of residence if stationary; or with the places they annually visit, if migratory. Those residing in cities are requested to give street and number.

20. Dentists who approve the objects of this Journal, and wish to give it more decided encouragement than by becoming subscribers for single copies, are requested to take several copies for private distribution; and also to obtain subscribers to the Journal.

As motives to induce the members of the profession to subscribe liberally for the work themselves, and to obtain subscriptions among their medical friends and others of their acquaintances, the Publishing Committee take the liberty of suggesting the following propositions:

1. There is no fraternity in the United States, of equal magnitude and importance, which has not some publication devoted to its interest.

2. The necessity of a general diffusion of knowledge in Dental Theory and Practice, if not felt by the profession, is at least deplored by the public.

3. There are many high-minded men of great knowledge and experience in our Art, who will gladly communicate the results of their observation, for the benefit of the younger members of the profession.

4. Such a work will have a tendency to expel from Dental Practice, the quackery which disgraces it, just in proportion as it dissipates ignorance on the subject from community at large.

5. It will give to distant parts of the country the benefit of all the Theoretical and Practical knowledge, which exists in our large cities, on the subject of operations on the teeth.

6. This publication will diffuse important kinds of information which is now local and limited, for want of a medium of publication.

7. It will associate in fraternal feeling, many who will be benefitted by the association.

8. This Journal will perpetuate those numerous Treatises on Dental Science and Practice, which are now nearly out of print; as well European as American.

9. If the members of the Profession, few in number compared with the patrons of other periodicals, do not subscribe liberally to the work, there is little probability that it can be long sustained.

10. The publication of such a Journal, will have the effect of giving dignity and importance to the general subject of Practical Dentistry; and thus result in solid advantage to each and all of its professors, as well as to community at large.

11. The state of the subscription list will indicate the scene of operation of the principal Dentists in the United States, and thus inform that portion of the profession who are not stationary in their labours, to what points their exertions may be most profitably directed.

12. The publication of the entire list of Practitioners in the United States, will have a tendency to check the exuberant influx of half educated aspirants, who imagine that the field of labour is but partially occupied, and thus expose themselves to ultimate disappointment.

In relation to the last of these propositions, it should be remarked, that although the number of dental practitioners in the United States, whose services are of any value to the public, is sufficiently limited, yet the persons who could convince the community of their real interests on the subject of their teeth, and induce them to employ the profession, must be differently educated from those whose abominable mal-practice has so long disgraced the profession, and imposed upon mankind.

It is to remedy this state of things as well in the practices of the profession as in the opinion of community, that the present work is proposed to be established—and we feel confident, that no Dental Operator who does not find it for his interest to perpetuate the present state of things, will refuse it his cordial support.

It was under the influence of this belief, that the Publishing Committee have issued this specimen number of the work, which they intend to forward by mail or otherwise, to every Dentist whose name and residence they can obtain in the United States and British provinces of North America. To enable them to effect this purpose, they solicit each and every individual in the profession, to forward to them, not only his own name and residence, but the name and residence of every professional brother with whom he may be acquainted in any state of the union. The letters should be post paid, for the obvious reason, that the price of postage will not be felt by the individual writer, but would be a burdensome item in the accounts of the publishers.

With these remarks, we submit to our professional brethren the important question, whether a dental magazine can be sustained in our country.

ELEAZAR PARMLY,
ELISHA BAKER, } PUBLISHING COMMITTEE.
SOLYMAN BROWN,

*New-York, June 1st,* 1839.

